# Ammonia-triggered disintegration of kappa-carrageenan hydrogel carrier for site-specific anti-inflammatory drug delivery

**DOI:** 10.3389/fbioe.2025.1676330

**Published:** 2026-01-06

**Authors:** Sachin Kumar, Priyank Purohit, Surbhi Panwar, Shivsharan Balbhim Kharatmal, Sachin Munjal, Magda H. Abdellattif, Chaitali Anil Thotange, Rachana Sambhaji mane

**Affiliations:** 1 Department of Pharmacy, Graphic Era Hill University Dehradun, Dehradun, India; 2 School of Pharmaceutical Sciences, Swami Rama Himalayan University, Dehradun, India; 3 Department of Pharmacology, Sinhgad Technical Education Society’s Smt. Kashibai Navale College of Pharmacy, Pune, Maharashtra, India; 4 Assistant Professor, Gastroenterology Medicine, Graphic Era Institute of Medical Sciences, Dehradun, India; 5 Chemistry Department, College of Sciences, University College of Taraba, Taif University, Taif, Saudi Arabia

**Keywords:** ammonia-sensitive hydrogel, ammoniasis, carrageenan, celecoxib, dual functional matrix, gel disintegration, IL-10, kappa carrageenan

## Abstract

Ammonia accumulation in tissues is increasingly recognized as a direct biochemical trigger of ammonia-induced inflammation, yet no therapeutic strategy currently exists that can selectively target this pathological condition while minimizing systemic toxicity. Addressing this critical gap, the present study introduces a first-of-its-kind kappa-carrageenan (KC)-based formulation engineered to respond selectively to ammonia-rich inflammatory environments while simultaneously exerting synergistic anti-inflammatory effects. The KC gel’s structural network exhibited pronounced disruption upon exposure to ammonium hydroxide, supported by physicochemical changes, the weakening of OH and SO_3_H absorption bands in FT-IR spectra, and optical microscopy-confirmed morphological alterations. Drug-release studies revealed highly accelerated celecoxib release (up to 86%) from NH_4_OH-treated gels compared to only 33% under normal physiological conditions, demonstrating strong ammonia-triggered responsiveness and high site-selective delivery. *In vivo* anti-inflammatory evaluation further confirmed enhanced therapeutic potency arising from the synergistic interaction between celecoxib and KC, while cell-line assays validated the formulation’s favorable safety profile. Although long-term stability and pharmacokinetic assessments are required for clinical translation, this study establishes KC as a dual-functional, smart, and ammonia-responsive system, offering a novel mechanistic framework for targeted, sustained, and effective treatment of ammonia-associated inflammatory disorders.

## 1 Introduction

**FIGURE 1:**
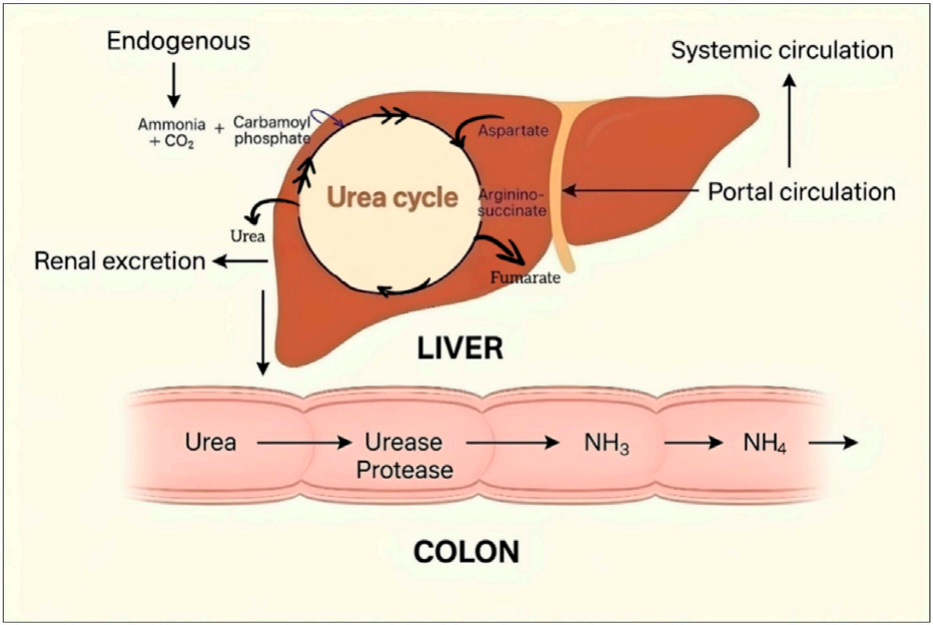
Schematic representation of ammonia metabolism and circulation between the colon and liver.

The burden of inflammatory disorders is an increasingly urgent question of global health concern, with high rates of affected patients, chronicity, and cross-system effects. According to the Global Burden of Disease (GBD) studies, the immune-mediated inflammatory diseases (IMIDs) including asthma, rheumatoid arthritis and inflammatory bowel disease (IBD) is estimated to cause around 0.60-0.70 % of all new cases worldwide per year (Wu et al., 2023; Liu et al., 2024). Although they are treated symptomatically, prolonged or unregulated inflammation is identified as a cause of a number of chronic illnesses, such as hepatic encephalopathy, systemic inflammatory conditions, and IBD (Clària et al., 2023). Therefore, identifying the causative agents and relevant biomarkers is essential for ensuring appropriate treatment. Among the newly emerging biochemical markers, the ammonium ion (NH_4_^+^), a naturally produced metabolic end product, has been increasingly associated with inflammation, especially in people with hepatic or renal dysfunction (Aldridge et al., 2015; Konturek et al., 1996). The production of ammonia occurs principally in the gut through microbial degradation of dietary proteins and nitrogenous materials (Richardson et al., 2013). Normally, under physiological conditions, the liver effectively clears it through the urea pathway to avoid systemic buildup (Figure 1) (Matsumoto et al., 2019). However, when conditions such as liver failure or intestinal dysbiosis are present, the body’s ability to eliminate ammonia becomes impaired. As a result, its concentration increases in the blood, and it can penetrate body organs such as the brain, liver, and kidneys. This overload of NH_4_^+^ may induce oxidative stress, mitochondrial redox dysfunction, and inflammatory signaling events that aggravate tissue dysfunction (Gallego-Durán et al., 2024).

**FIGURE 2:**
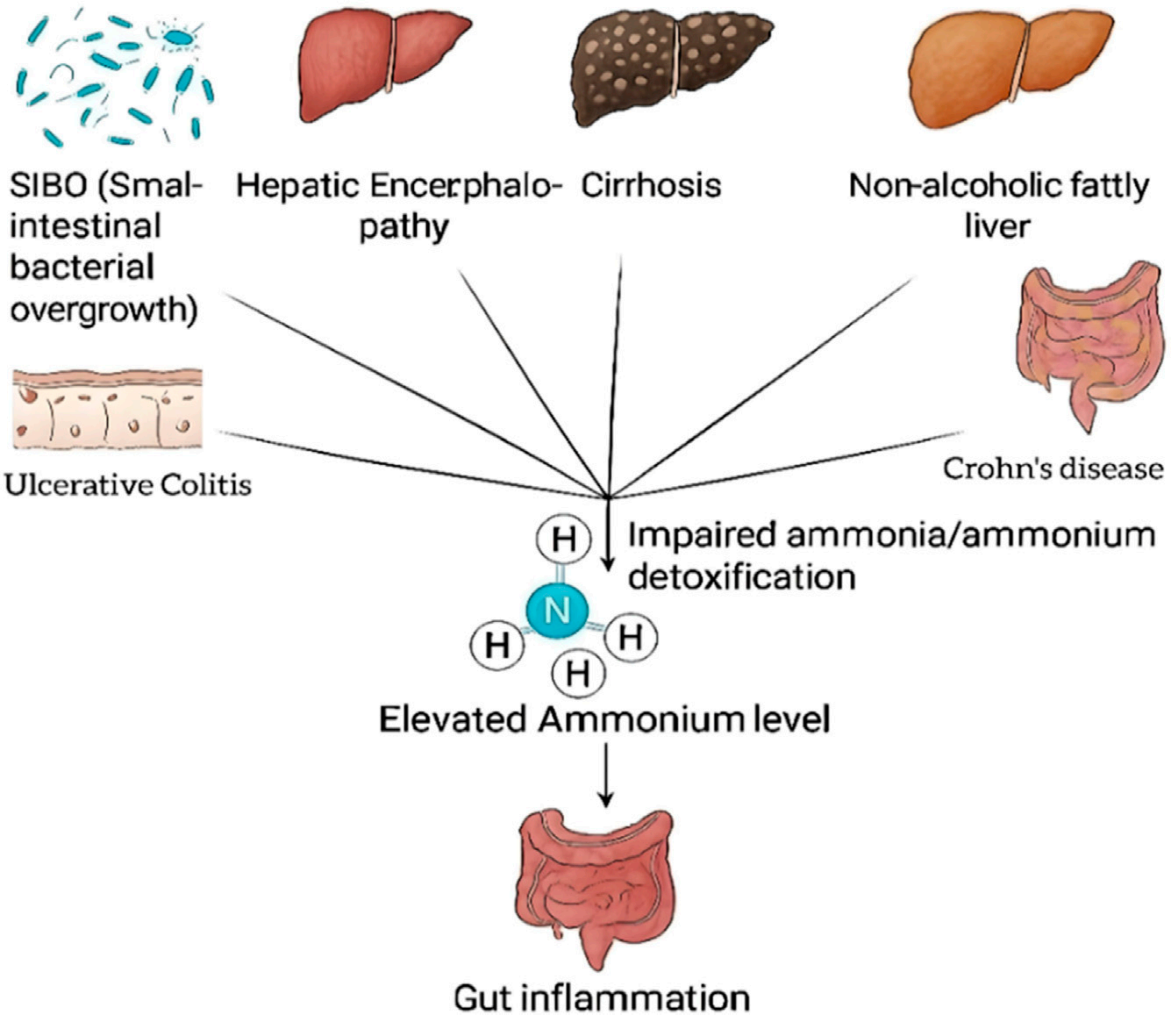
Pathophysiological links between systemic ammonia accumulation and inflammation.

Ammonia has been implicated in numerous inflammatory conditions, including hepatic encephalopathy, small intestinal bacterial overgrowth (SIBO), non-alcoholic fatty liver disease (NAFLD), and IBD (Figure 2). These conditions are mainly mediated through the loss of immune homeostasis and the induction of pro-inflammatory cytokine release in the bowel (Walker, 2014).

Effective management of ammonia-induced pathophysiological conditions necessitates therapeutic strategies that address both elevated ammonia levels and the associated inflammatory responses. Lipid-soluble antioxidants such as lipoic acid and N-acetylcysteine have been reported to attenuate ammonia-induced oxidative stress and inflammation in astrocytic cells, indicating the feasibility of antioxidant-based therapies for managing ammonia-induced neuroinflammation (Santos et al., 2015). Similarly, dietary supplements such as betaine have demonstrated efficacy in alleviating ammonia-induced inflammation and apoptosis across various experimental models (Chen et al., 2022; Ntuli and Shawcross, 2024). Conventional treatment protocols primarily include antibiotics to reduce intestinal bacterial load and thereby decrease systemic ammonia levels (Lauritano et al., 2007; Liu et al., 2018). However, probiotics offer a promising alternative by modulating gut microbiota to enhance ammonia detoxification without the adverse effects commonly associated with antibiotics (Meng et al., 2019). Several studies indicate that strategies aimed at reducing systemic ammonia concentrations can effectively mitigate its neurotoxic and inflammatory effects. For instance, oral adsorbents such as AST-120 have been shown to reduce intestinal ammonia absorption, thereby alleviating hepatic encephalopathy symptoms and potentially protecting against gut inflammation (Bosoi et al., 2011). Experimental evidence further supports that prebiotic and probiotic interventions help modulate the gut microbiome, lowering ammonia levels and mitigating inflammatory responses (Ahluwalia et al., 2016; Bajaj et al., 2024; Shen et al., 2015). Similarly, microbiota-targeted interventions such as the systemically acting antibiotic rifaximin have significantly reduced ammonia-mediated neurocognitive impairment in cirrhotic patients, highlighting the therapeutic importance of gut microbiota modulation (Yukawa‐Muto et al., 2022). Dietary modulation remains another key aspect of ammonia management. Low-protein diets have been shown to reduce endogenous ammonia production, maintain gut integrity, and suppress inflammation (Li et al., 2022). Additionally, nutritional supplements such as selenium exert neuroprotective effects by improving gut microbial composition and reducing oxidative stress in ammonia-induced conditions. Elevated ammonia levels disrupt intestinal epithelial tight junctions, leading to increased gut permeability and inflammation; therefore, maintaining gut barrier function is critical (Liu et al., 2022; Ricci et al., 2002; Görg et al., 2009; Vaziri et al., 2012). Therapeutic approaches focusing on restoring intestinal barrier integrity, including pharmacobiotics or anti-inflammatory compounds, have shown potential in this regard. The use of non-steroidal anti-inflammatory drugs (NSAIDs) has also emerged as a promising approach for mitigating ammonia-induced inflammation. NSAIDs inhibit cyclooxygenase (COX) enzymes, thereby reducing the synthesis of pro-inflammatory prostaglandins responsible for propagating inflammatory cascades (Vane and Botting, 1998). Indomethacin, in particular, can prevent ammonia-induced brain edema, underscoring its potential in alleviating neuronal inflammation (Chung et al., 2001). Studies on gastric mucosal cells further reveal that NSAIDs inhibit ammonia-induced vacuolation, contributing to cellular protection under inflammatory stress (Cauli et al., 2009). Moreover, NSAIDs exhibit neuroprotective effects in models of acute hyperammonaemia, mitigating both edema and apoptosis induced by ammonia exposure (Wright and Jalan, 2007). This highlights a multifaceted role for NSAIDs, wherein they attenuate inflammation and concurrently prevent secondary neuronal damage associated with ammonia toxicity. In summary, the current therapeutic strategies for ammonia-induced inflammation primarily focus on reducing systemic ammonia levels or mitigating inflammatory responses through antioxidants, antibiotics, probiotics, or dietary modulation. Although approaches such as probiotic therapy, low-protein diets, selenium supplementation, and NSAID administration have shown potential in lowering ammonia toxicity and inflammation, these interventions often lack targeted delivery and may cause systemic side effects, particularly with chronic NSAID use. Notably, no study has yet reported an ammonia-responsive polymer-based delivery system capable of site-specific NSAID release.

**FIGURE 3:**
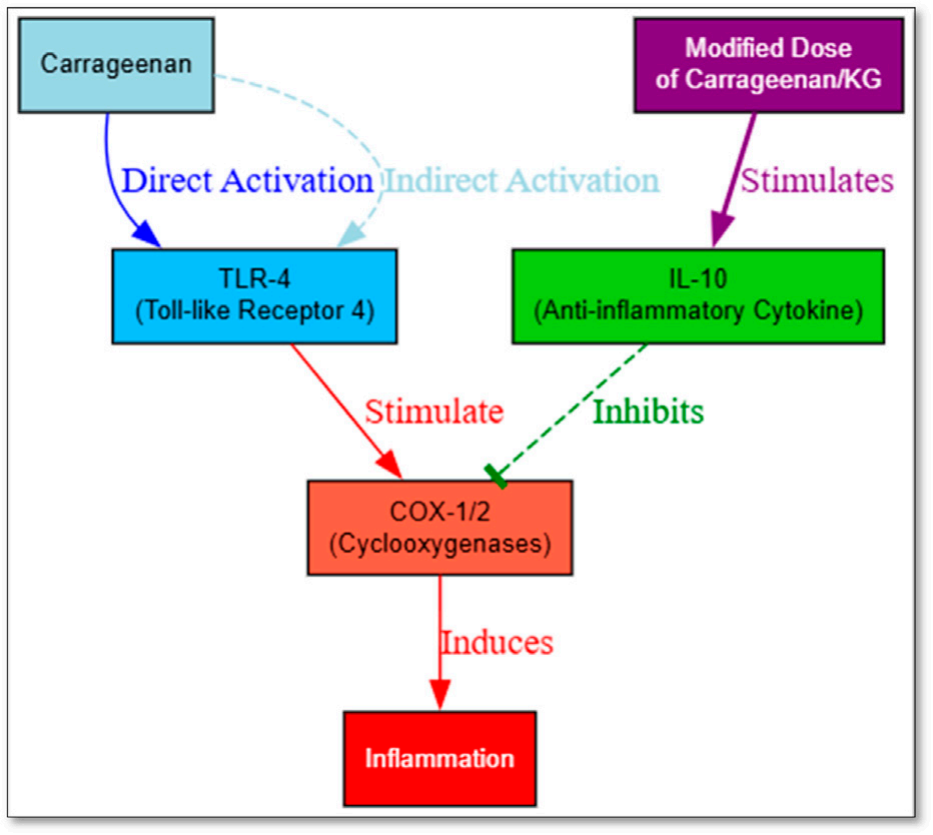
Schematic representation of carrageenan-induced inflammation and IL-10-mediated regulation.

The structural characteristics of carrageenan, particularly its high sulfate content, contribute to its bioactivity. These features enable carrageenan to interact effectively within ammonia-rich environments, making it a suitable polymer for targeted anti-inflammatory applications (Pradhan and Ki, 2023; Kumar et al., 2025). At the mechanistic level, it triggers key inflammatory pathways, including TLR-4/NF-kB, reactive oxygen species (ROS), and AP-1, and is known to induce the production of cytokines and prostaglandins (Kany et al., 2019; Vazquez et al., 2015). Among these mediators, IL-1 B, IL 6, IL 8, and TNF-alpha are key drivers of inflammatory process. The same cytokines induce the expression of cyclooxygenase-2 (COX-2), which is a crucial enzyme in the biosynthesis of prostaglandins and transmission of pain (McKim et al., 2015) (Figure 3). However, according to other reports, The carrageenan does not appear to directly bind to the TLR-4, as demonstrated in a study by Yao et al. (2022) which used a TLR-4/MD-2/CD14/NF-kappa B/SEAP reporter system in HEK293 cells (Yao et al., 2022). Interestingly, structurally modified carrageenan derivatives, such as oligosaccharides or poligeenan, have shown anti-inflammatory effects, and it is important to note the specificity of the immunological responses associated with carrageenan (Yermak et al., 2012). Specifically, it has also been suggested that kappa-carrageenan (KC) increases the synthesis of anti-inflammatory cytokine interleukin-10 (IL-10) in a dose-dependent manner.

Among the carrageenan family, kappa-carrageenan (KC) exhibits a strong gelling capacity, by forming stiff, brittle networks through extensive aggregation of its double-helical chains, in contrast to the soft and elastic gels formed by iota-carrageenan (IC) and other carrageenan types (Herm and ansson, 1989; Watase et al., 1990; Mirzaei et al., 2023; Hilliou, 2021). Hence, the dual functionality of such a polymer—both sensing ammonia and exerting an anti-inflammatory effect—was attempted here to offer a synergistic and safer alternative to existing treatments. The present work introduces this novel concept, providing preliminary evidence of efficacy, though further comprehensive investigations are warranted to optimize its therapeutic performance and safety.

## 2 Materials and methods

KC was procured from Marine Hydrocolloids, and celecoxib was obtained from Cipla Pharmaceuticals Ltd. (India). Analytical-grade ammonium hydroxide (NH_4_OH), potassium chloride (KCl), and other chemicals were obtained from Merck India. Sampling was performed using an IR Spectrometer (Spectrum 2 model with serial number 89258) with the KBr pellet method, and the data analysis program was NIOS2. UV–visible spectrophotometric measurements were performed using a UV–VIS spectrophotometer (UV-1900 Series, Model UV-1900i, Serial No. A12536082965, Shimadzu Corporation, Japan).

All solutions were prepared using deionized double-distilled water. Male Wistar albino rats (180–220 g) were used for *in vivo* anti-inflammatory studies, conducted in accordance with CPCSEA guidelines and approved by the Institutional Animal Ethics Committee (IAEC No. SKNCOP/IAEC-159–27/2023). Instruments used included a digital rotational viscometer (LABMAN, LMDV-60), a conductivity meter (Hanna EC 215), and a binocular microscope (MV-LED) with dual lenses. Images were captured using Capture Pro software at magnifications ranging from 100× to 2000×. All equipment and materials were used following calibrated, precise, and standardized protocols.

### 2.1 Methodology

#### 2.1.1 Preparation of celecoxib loaded KC gel

To optimize KC gelation, both temperature and concentration were systematically varied. A 1% w/v KC solution was heated to 60 °C in a magnetic stirrer at a rotation of 500 per minute and slowly cooled to 4 °C, forming a clear, self-supporting gel and confirming the role of thermal activation. In contrast, at room temperature (∼24 °C), gelation was not observed below 4% w/v; a firm gel developed only at 5% w/v, indicating a concentration threshold for spontaneous gelation. These findings established two optimized conditions for KC gel formation: thermal gelation at 1% w/v with heating and concentration-induced gelation at 5% w/v at room temperature. Therefore, the 1% w/v KC solution method under the heating condition with 500 rpm was used and subjected to further evaluation.

#### 2.1.2 Evaluation of ammonia responsiveness on KC gel

The assessment of ammonia sensitivity was conducted by measuring the conductivity of 1% KC gels after the addition of increasing quantities of NH_4_OH (100–900 µL) to detect ion disruption and evaluate the integrity of the matrix. Gel–sol transition was also determined by measuring conductivity as the solution was heated in a controlled manner between 19.5 °C and 60 °C, and the structural changes in blank and NH_4_OH-treated KC gels were examined via FT-IR analysis. Pelletized samples of dried gel were blended with potassium bromide (KBr) and formed into pellets. An FT-IR spectrophotometer was used to record spectra in the range of 500–4000 cm ^-1^.

#### 2.1.3 Quantitative evaluation of conductivity and viscosity parameters

To study the ionic behavior and thermosensitive properties, the conductivity of the blank and NH_4_OH-treated KC gels was measured at a temperature range of 21 °C–81 °C using a calibrated digital conductivity probe (Hanna EC 215). Temperature-dependent ionic transitions were monitored by recording real-time measurements at fixed intervals. Further assessment of conductivity was carried out upon addition of incremental quantities of NH_4_OH (100–900 µL) to evaluate the structural modification by ammonia. Viscosity measurement was carried out using a digital viscometer (LABMAN LMDV-60) at 10 °C–40 °C-controlled shear. Each measurement was performed in triplicate under the same ambient conditions.

#### 2.1.4 Microscopic analysis of gel morphology

The samples of both blank and NH_4_OH-treated KC gel were observed under a microscope to determine their structural integrity. Glass slides with small gel samples were used under an optical microscope (binocular microscope) at ×4 magnification. Capture Pro software-based images were used to analyze the morphological changes, such as network disruption or gel loosening resulting from NH_4_OH exposure, were assessed.

#### 2.1.5 Drug diffusion dynamics and kinetic modeling approaches

The drug release of blank KC gel and NH_4_OH-treated KC gel loaded with celecoxib was investigated using a Franz diffusion cell apparatus. Approximately 1 g (containing 10 mg of drug) of each gel was introduced into the donor chamber. The receptor cell hutch was loaded with phosphate buffer to act as the dissolution medium. At predetermined intervals (0–180 min), 1 mL samples were withdrawn and replaced with fresh buffer. Absorbance was measured at 254 nm using a UV–visible spectrophotometer, and cumulative percentage release (Q_t_) was calculated from the calibration curve. To elucidate the mechanism of drug release, the obtained release data (Q_t_ vs. t) were fitted to various kinetic models, namely, zero-order, first-order, Higuchi, and Korsmeyer–Peppas models. The mathematical relationships and mechanisms of these models are summarized in Supplementary Table S7. For each model, the rate constant (k), release exponent (n) (only for the Korsmeyer–Peppas model), and correlation coefficient (*r* ^2^) were determined by nonlinear regression analysis using GraphPad Prism 10.0 software. The model with the highest *r* ^2^ value was considered to provide the best description of the drug release mechanism.

#### 2.1.6 *In vivo* anti-inflammatory study

The Institutional Animal Ethics Committee (IAEC) approved all experimental protocols and procedures (approval no.: SKNCOP/IAEC-159–27/2023). The carrageenan-induced paw edema model was employed to evaluate the anti-inflammatory activity of KC, celecoxib, and their combination in male Sprague–Dawley rats. Animals were divided into four groups, with six rats in each group (n = 6). The study used animals (age: 12–15 weeks; weight: 200–225 g) procured from Crystal Biological Solution, Pune, and maintained under standard conditions (21 °C, 12:12-h light–dark cycle, 40%–60% humidity) with free access to food and water. Approximately 0.1 mL of the inducing agent (a 1% carrageenan suspension in 2% gum acacia) was injected into animals via the subplantar route, and acute inflammation was determined 1 h after treatment. The volume of paw edema was measured at 0, 3, and 5 h using a digital plethysmometer after administering KC, celecoxib, and a blend of KC and celecoxib in different groups of animals.

#### 2.1.7 *In silico* molecular docking analysis

The binding affinities of KC and KC blend Celecoxib, was performed toward inflammatory signaling targets like TLR4, NF-κB, and JAK/STAT.

### 2.2 Statistical analysis

All data from the *in vivo* experiments were expressed as the mean ± standard error of the mean (SEM). Statistical comparisons between more than two groups were performed using one-way analysis of variance (ANOVA), followed by Dunnett’s *post hoc* test for multiple comparisons against the control group. For comparisons between two groups, Student’s t-test was applied where appropriate. A *p*-value of <0.05 was considered statistically significant. Statistical analysis and graph plotting were performed using Sigma Stat software (Jandel Scientific, Germany).

## 3 Results and discussion

The KC gel was prepared first via the thermo-reversible sol–gel transition, which solidifies at low temperatures to obtain a gel with optimal consistency and stability as it has been reported that K^+^ kappa carrageenan forms a stiffer and more stable gel (Wang et al., 2018). The K^+^ was confirmed by the Atomic Absorption Spectroscopy (AAS) with more than 99%, (Graph is provided in Supplementary Figure S1 of Supplementary Material), so the expected gel was anticipated with the high stability and consistency. The stability of the gel was evaluated at room temperature (25 °C) for 15 days, during which its viscosity (640–670 mPa·s) and conductivity (9.0 ± 1.2 mS/cm) remained nearly constant, indicating structural integrity (Kumar et al., 2025). Beyond this period, slight water separation was observed. Under refrigerated conditions (4 °C), however, the gel retained its stability for up to 2 months. Although long-term stability remains a challenge for the present formulation, the incorporation of antimicrobial agents and ion-stabilizing additives has been found to further enhance its shelf life (Cappa et al., 2024).

### 3.1 Evaluation of the gel

**FIGURE 4:**
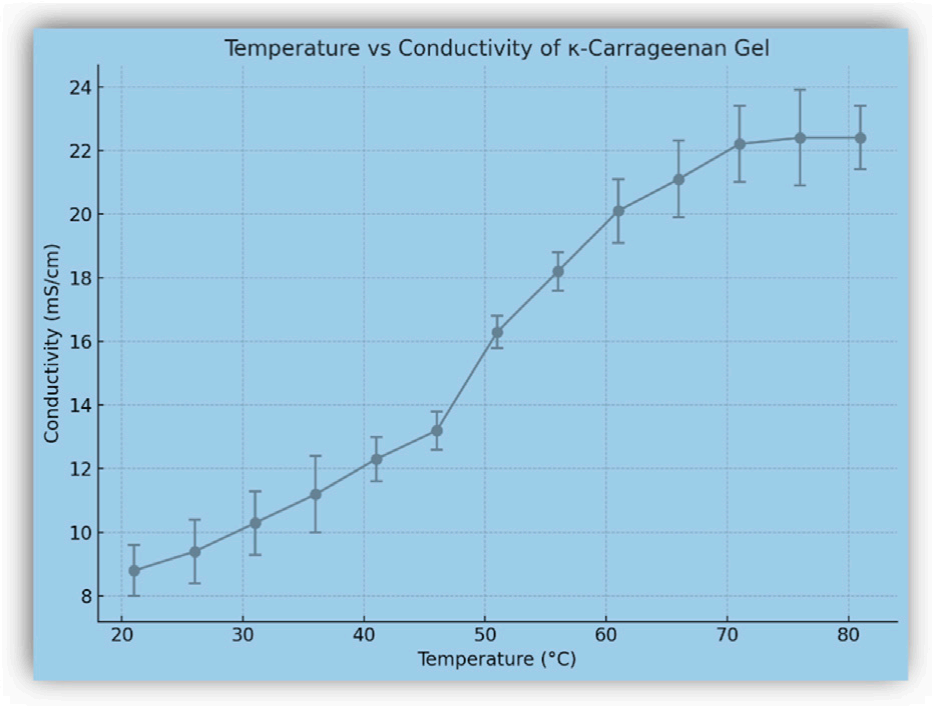
Conductivity of KC gel as a function of temperature.

*In situ* gel formation has emerged as a promising alternative for targeted delivery, and detailed investigations in this area are currently being conducted by our group (Garg et al., 2024; Vigani et al., 2019). The primary focus of the present invention is to elucidate the ionic networking within the gel core under specific conditions and its corresponding initial pharmacological response. The biological evaluation of the compound was carried out using an *in vitro* cell assay as per the reported protocol by our group (Mobarak et al., 2015) using the LLC-MK2 cell line as a model for healthy cells. The observed IC_50_ value of 741.23 μg/mL indicates a non-toxic nature of κ-carrageenan (% inhibition values provided in Supplementary Table S1 of Supplementary Material). Furthermore, considering that κ-carrageenan is a well-recognized food-grade polysaccharide, its safety at normal doses is well established. Conductivity and temperature-based analysis (values provided in Supplementary Table S2 of Supplementary Material) (Kailkhura et al., 2024) were also performed to determine the ionic accumulation pattern of the gel during continuous heating between 21 °C and 81 °C (Figure 4), in triplicate. First, conductivity increased gradually with temperature, the result of increased ionic mobility. Beyond 50 °C, conductivity sharply increased and reached the maximum at 75 °C, indicating a gel–sol phase transition. At this point, this sharp increase can be correlated with the destruction of the gel matrix and the promotion of ion migration in the sol state. The fact that conductivity remained plateaued over and above 75 °C indicated the total breakdown of the network and stable ionic migration. This thermal property confirms that KC is highly sensitive toward temperature and possesses a sharp phase transition. The temperature and conductivity patterns were associated with the physical observation and corresponding phase change

### 3.2 Evaluation of gel disintegration behavior in ammonium solution

**FIGURE 5:**
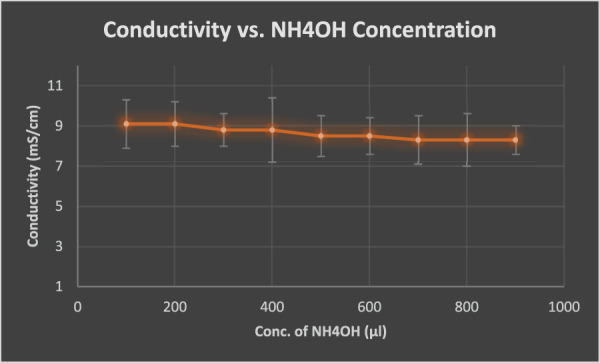
Change in conductivity with increasing NH_4_OH concentration.

The fact, that it forms stable gels at low concentrations and moderate temperatures, could be favorable for the therapeutic-delivery in ammonia-induced inflammatory condition. Ammonia is known to enhance inflammation and is available in the form of ammonium hydroxide, depending on the pH of the body. Further experiments were conducted to clarify the integrity of gels by comparing Figure 4 and the ionic properties under ammonia-actuated conditions. The gel-to-sol temperature range was physically confirmed by observing the gel liquefaction process. To mimic inflammatory conditions, conditions of conductivity were measured when NH_4_OH was added to KC gel gradually (values provided in Supplementary Table S3 of Supplementary Material). With the addition of NH_4_OH, a decrease in ion mobility was observed as the value decreased to 8.3 mS/cm (compared to the initial 9.1 mS/cm) (Figure 5). This is possibly due to the interaction of ammonium ions, which align and disrupt the electrostatic structure of the gel matrix (Lopes et al., 2012).

The analysis of viscosity also indicated a significant reduction with the NH_4_OH exposure. At 10 °C, the viscosity of blank KC gel was measured to be 643.2 mPa·s, whereas NH_4_OH-treated gel demonstrated a value of 622.7 mPa·s. This decreased to 127.8 mPa·s at 40 °C, indicating that the gel matrix had broken down and a gel-to-sol conversion has occurred (see Table 1; values have been taken in triplicate).

**TABLE 1:** Viscosity of KC gel with and without NH_4_OH at different temperatures.

Temperature (^o^C)	Viscosity (mPa-s)	Viscosity (mPa-s) of gel with NH4OH
10	643.23 ± 2.3	622.66 ± 1.3
40	584.38 ± 1.8	127.80 ± 3.2

**FIGURE 6:**
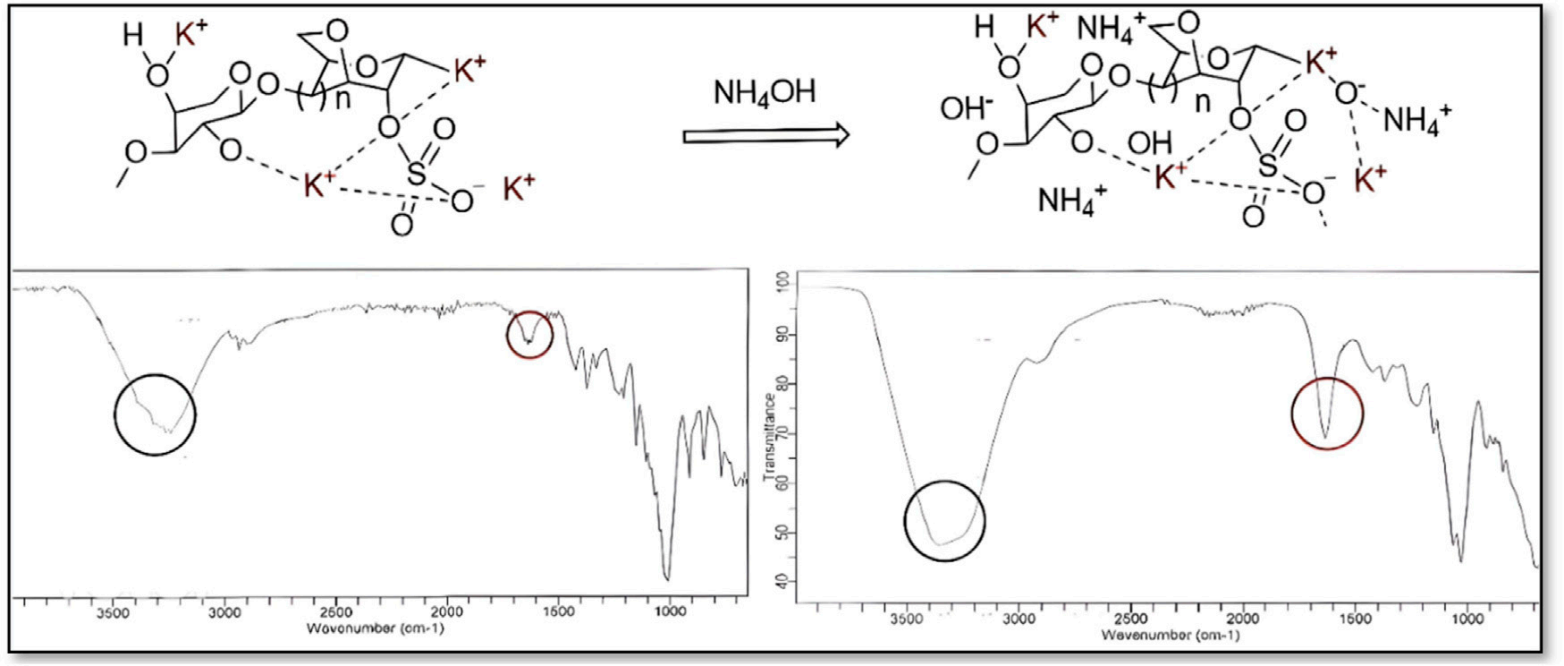
FT-IR spectra of blank and NH_4_OH-treated kappa-carrageenan gels.

The FT-IR analysis revealed a broadening of the OH stretching bands and a reduction in the intensity of the SO_3_H peaks in NH_4_OH-treated κ-carrageenan (KC) gels (Bhatt et al., 2024a), indicating the involvement of hydrogen bonding and the disruption of sulfate ion-mediated cross-links within the KC network (Figure 6). Such spectral alterations and viscosity results shows the destabilization of gel under an ammonia-rich environment. An overview of the spectral shifts, along with detailed interpretations, is provided in the graph and values shown in Supplementary Figure S2 and Supplementary Table S4 of Supplementary Material.

**FIGURE 7:**
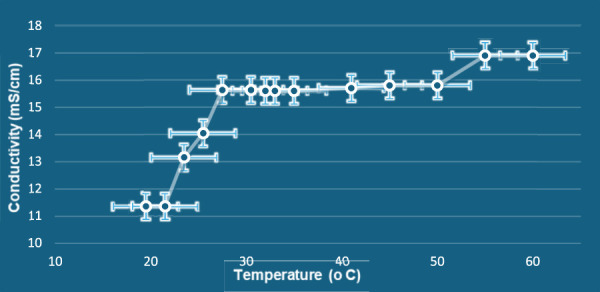
Graph between temperature and conductivity of NH_4_OH-treated KC gel.

The effect of NH_4_OH on the temperature-dependent conductivity behavior was evaluated (Figure 7). A sharp increase in conductivity was observed between 23.5 °C and 27.5 °C, indicating an earlier onset of the gel–sol transition in NH_4_OH-treated gels compared to the blank, which exhibited the transition near 60 °C. This suggests that NH_4_OH weakens the intermolecular interactions within the κ-carrageenan network, thereby facilitating an earlier disruption of the gel structure.

**FIGURE 8:**
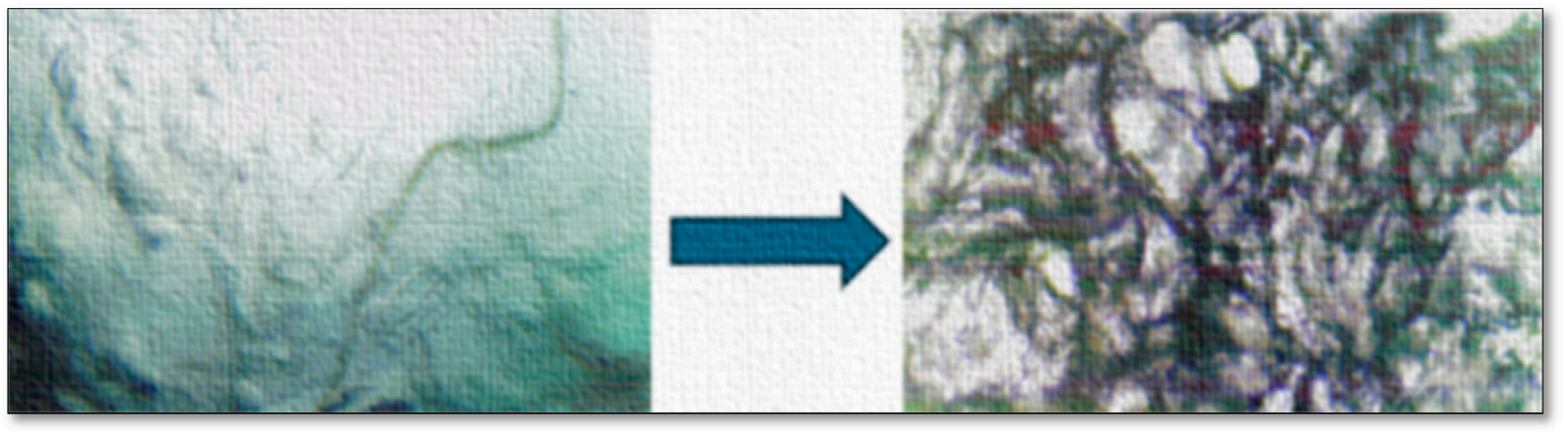
Optical microscopy (4 ×) of blank KC gel (left) and NH_4_OH-treated gel (right) showing visible matrix disruption.

The effect of ammonium ion or ammonia was also analyzed via microscopic image captured before and after NH_4_OH addition (Figure 8). The analysis of the images revealed significant structural alterations at the micro-level. Notably, the gel matrix exhibited a pronounced loosening of its fibrous network, along with initial signs of disintegration and fragmentation. These morphological changes suggest a disruption of the intermolecular interactions, particularly ionic crosslinking and hydrogen bonding that are critical for the stability and rigidity of the carrageenan gel framework. The observed microstructural degradation aligns coherently with the earlier conductivity and viscosity data, which indicated a decrease in network integrity. The visual evidence thus substantiates the hypothesis that NH_4_^+^ ions interfere with the native gelation behavior of KC, possibly by replacing stabilizing cations (such as K^+^ or Ca^2+^) or altering the electrostatic environment, thereby weakening the overall gel structure.

**FIGURE 9:**
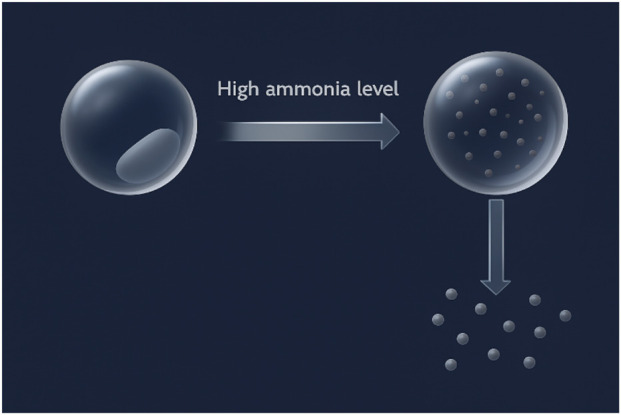
Illustration of site-specific KC gel disintegration triggered by elevated ammonia levels.

The gel-destabilizing activity of ammonium ions also occurred with ammonium sulfate, indicating that NH_4_^+^ is the main destabilizing species. The sharp decrease in transition temperature (from 60 °C to 25 °C) favors the contention that NH_4_OH destabilizes the K^+^ sulfate coordination responsible for stabilizing gels. This characteristic of ammonia-based disintegration allows drugs aimed at the localization of anti-inflammatory drugs. According to Figure 9, KC gel is erosion-resistant in the normal physiological tissues but degrades in the ammonia-loaded chicken tissues, providing regulated release of drugs (Bhatt et al., 2024b).

The results are in line with the fact that KC gel has both structural stability under physiological conditions and selective disintegration in response to ammonia. These two characteristics enable the design of responsive, inflammation-specific drug delivery with the potential to allow systemic minimization and enhance therapeutic specificity. Keeping these significant findings in mind, the gel was explored as a carrier for a well-stabilized anti-inflammatory drug, with the potential for precise targeting of ammoniarich tissues.

The KC gel was treated with celecoxib during gel formation to entrap the drug inside the core of the gel. The UV visibility of celecoxib, unlike KC, clearly confirms the presence of the drug inside the gel. The drug entrapment study was conducted via UV–visible spectrophotometry at 254 nm (nanometers), with the amount of celecoxib determined from a calibration curve of absorption vs. concentration (calibration data are provided in Supplementary Table S5; Supplementary Table S4 of Supplementary Material). Entrapment efficiency was 52%, with 5.2 mg of celecoxib retained in the gel. Unentrapped drug was removed via centrifugation, with the supernatant collected, and the gel pellet was washed with buffer to eliminate any free drug prior to analysis (Ban et al., 2020).

### 3.3 Drug release study

**FIGURE 10:**
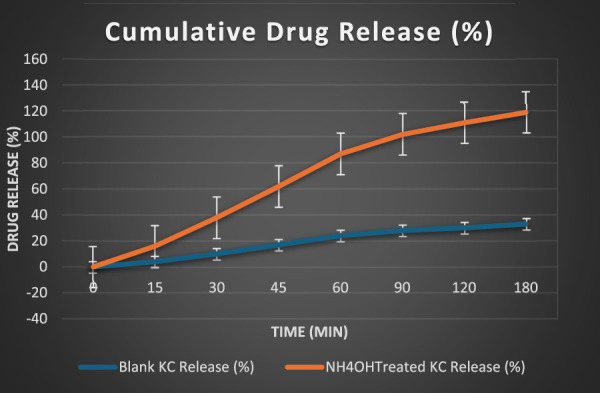
Cumulative drug release from blank and NH_4_OH-treated KC gels.

The entrapped gel was evaluated for *in vitro* release using a Franz diffusion cell to assess how celecoxib was released from both KC formulations (with or without NH_4_OH) over a 3-h period (180 min). The donor compartment contained celecoxib-entrapped blank KC and NH_4_OH-treated gel, while the phosphate buffer (pH 7.4) was added to the receptor chamber. Samples were periodically collected for UV spectrophotometric measurement at 254 nm (Figure 10). The NH_4_OH-treated KC gel revealed a much faster and greater cumulative release profile, reaching 86% release at 180 min (relevant data are provided in Supplementary Table S6 of Supplementary Material). Conversely, under the same circumstances, the blank KC gel displayed a longer prolonged release, only attaining 33%.

### 3.4 Kinetic analysis of drug release

**FIGURE 11:**
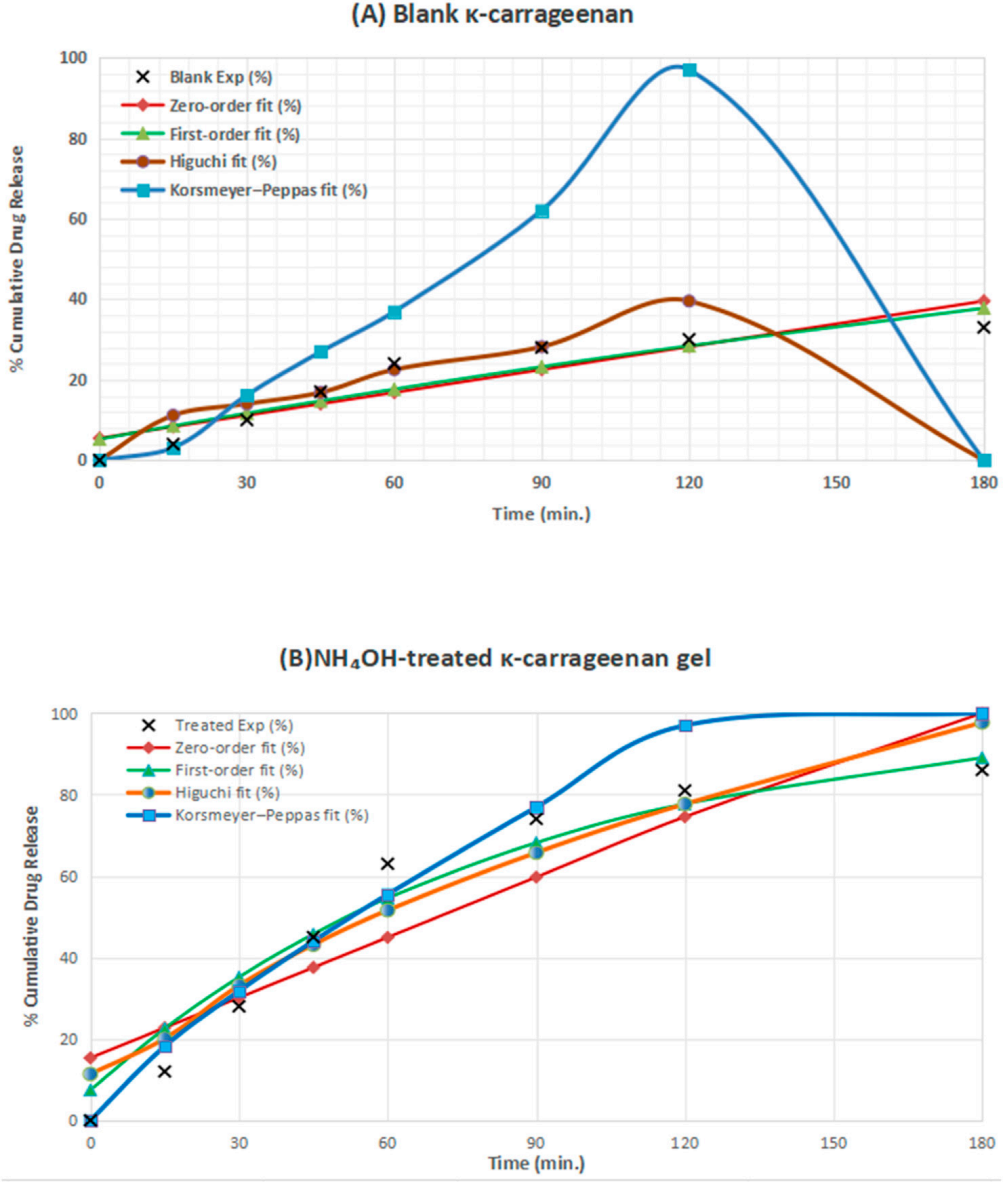
Fitted kinetic models for *in vitro* celecoxib release from κ-carrageenan gels. **(A)** Blank KC gel showing experimental data and model fits (zero order, first order, Higuchi, and Korsmeyer–Peppas). **(B)** NH_4_OH-treated KC gel showing enhanced release kinetics consistent with the Korsmeyer–Peppas (non-Fickian) mechanism.

The release data were fitted to the four classical kinetic models, and the corresponding parameters (*k*, *n*, and *r* ^*2*^) (all the data are provided in Supplementary Tables S7, S8 of Supplementary Material) and the corresponding model-fitted curves are illustrated in Figure 11. Panel A of Figure 11 depicts the experimental and fitted profiles for the blank KC gel, whereas Panel B shows the profiles for the NH_4_OH-treated gel. The close agreement between experimental and predicted values confirms the reliability of the applied kinetic models.

The blank KC gel followed the Higuchi model (*r* ^2^ = 0.991), indicating diffusion-controlled release through the polymer network. In contrast, the NH_4_OH-treated gel fitted best to the Korsmeyer–Peppas model (*r* ^2^ = 0.974, *n* = 0.68), suggesting anomalous (non-Fickian) diffusion governed by both polymer relaxation and diffusion. The higher *k* in the treated gel reflects ammonia-induced disruption of ionic cross-links, consistent with FT-IR and conductivity findings (detailed data are provided in Supplementary Table S8 of Supplementary Material.) These observations confirm that ammonia triggers loosening of the κ-carrageenan matrix, accelerating celecoxib diffusion and producing a site-responsive burst release. This dual behavior highlights the potential of ammonia-sensitive KC gels for targeted and controlled drug delivery, particularly under pathological conditions marked by elevated ammonia concentrations, such as hepatic encephalopathy and inflammatory bowel disease. Subsequently, the site-specific drug-release phenomenon was investigated by evaluating the in vivo anti-inflammatory effect of the drug, both in the presence and absence of the KC gel.

### 3.5 *In vivo* anti-inflammatory evaluation of the drug-loaded gel

To evaluate the *in vivo* anti-inflammatory efficacy of KC, celecoxib, and celecoxib blended kappa carrageenan, rat paw edema-induced models were utilized. Edema volume was measured at 3- and 5-h post-induction to assess both immediate and sustained inflammatory responses (Hariyadi et al., 2021; Cicinskas et al., 2019). As shown in Table 2, the reference drug, celecoxib (50 mg/kg), exhibited a significant reduction in paw edema (*p* < 0.001), validating the experimental model.

**TABLE 2:** Percentage inhibition of carrageenan-induced paw edema by KC and celecoxib.

Treatment	Dose (mg/kg)	% Inhibition at 3 h	% Inhibition at 5 h
Control	0	0	0
Celecoxib	50	77.8 ± 0.5 ***	75.0 ± 0.2 ***
KC	50	55.5 ± 0.2 ***	57.5 ± 0.4 ***
KC + celecoxib	50	79.3 ± 0.1	75.3 ± 0.1

^a^ Values are presented as the mean ± SEM. Significance: *p < 0.05, **p < 0.01, **p < 0.001 vs. *control. Analysis: one-way ANOVA, followed by Dunnett’s post hoc test*.

The superior anti-inflammatory efficacy of celecoxib with KC at a dose of 50 mg/kg may be linked to its unique structural configuration. KC possesses a single sulfate group per disaccharide unit, which likely results in a weaker interaction with Toll-like receptor 4 (TLR-4), a key mediator of inflammatory signaling. Reduced TLR-4 stimulation correlates with lower expression of pro-inflammatory cytokines. In parallel, both carrageenan types appear to activate anti-inflammatory regulatory mechanisms. KC was observed to enhance the release of interleukin-10 (IL-10), a cytokine that modulates immune responses through the JAK1/STAT3 signaling cascade. This pathway not only amplifies IL-10 expression but also suppresses T-cell activation and downregulates NF-κB-dependent expression of TNF-α, IL-1β, and IL-6 (detailed docking data are provided in Supplementary Table S9 of Supplementary Material).

KC’s dual capacity for ammonia responsiveness and targeted drug release, with synergistic anti-inflammatory action, positions it as an ideal candidate for developing intelligent, site-specific delivery platforms aimed at treating ammonia-associated inflammation. These conclusions are further substantiated by molecular docking studies, which confirm KC’s lower affinity for TLR-4 and enhanced interaction with IL-10 regulatory proteins compared to IC. These findings not only validate the anti-inflammatory utility of KC but also establish its dual functional potential as both a structural carrier and an immunomodulatory agent, making it uniquely suited for inflammation-driven diseases with localized ammonia accumulation. The *in vivo* studies conducted thus far have demonstrated anti-inflammatory activity. Future investigations must specifically focus on ammonia-induced inflammation models to advance the therapeutic candidate toward clinical translation.

## 4 Conclusion

This study establishes KC as a promising dual-functional biomaterial exhibiting synergistic anti-inflammatory potential and ammonia-responsive drug release behavior. The disruption of the KC gel network in the presence of ammonium hydroxide was confirmed by FT-IR and morphological analyses, leading to enhanced celecoxib release and improved therapeutic efficacy under ammonia-induced inflammatory conditions. However, certain limitations, such as microbial growth, partial de-gelling over time, and the potential for inflammation at higher doses, highlight the need for further optimization. Future research will focus on improving the formulation’s long-term stability through *in situ* gel systems or the incorporation of antimicrobial and antioxidant agents. Additionally, systematic studies on safety–dose relationships and targeted delivery parameters will be crucial to fully establish its therapeutic applicability. Overall, this investigation provides an essential foundation for developing an intelligent, ammonia-sensitive, and synergistically active anti-inflammatory platform, particularly relevant to ammonia-associated inflammatory disorders that contribute to severe pathological conditions.

## Data Availability

The datasets presented in this study can be found in online repositories. The names of the repository/repositories and accession number(s) can be found in the article/Supplementary Material.
