# Supplementary material for: Ammonia-triggered disintegration of kappa-carrageenan hydrogel carrier for site-specific anti-inflammatory drug delivery
Source: Front Bioeng Biotechnol. 2026 Jan 6;13:1676330. doi: 10.3389/fbioe.2025.1676330 (PMC12815811; doi:10.3389/fbioe.2025.1676330)
Supplement: Supplementary file 1 [file DataSheet1.docx]

**Ammonia-Triggered Disintegration of Kappa-Carrageenan Hydrogel carrier for Site-Specific Anti-inflammatory Drug Delivery**

Sachin Kumar^a^, Priyank Purohit^b**^, Surbhi Panwar^a^, Shivsharan Balbhim Kharatmal ^c^ Sachin Munjal^d^, and Magda H. Abdellattif ^e^ Chaitali Anil Thotange^c^, Rachana Sambhaji mane ^c^.

^a^Department of Pharmacy, Graphic Era Hill University Dehradun, Dehradun, 28008, India, ^b^School of Pharmaceutical Sciences, Swami Rama Himalayan University, Jolly Grant, Dehradun, 248016. ^c^Department of Pharmacology, Sinhgad Technical Education Society’s Smt. Kashibai Navale College of Pharmacy, Kondhwa (Bk.), Pune 411 048, Maharashtra, India. ^d^ Assistant Professor, Gastroenterology Medicine, Graphic Era Institute of Medical Sciences, Dehradun. India. ^e^Chemistry Department, College of Sciences, University College of Taraba, Taif University, P. O. Box 11099, Taif 21944, Saudi Arabia

**Corresponding author:**

**Dr Priyank Purohit**

**Email:**  [**prpurohit@srhu.edu.in**](mailto:%20prpurohit@srhu.edu.in)


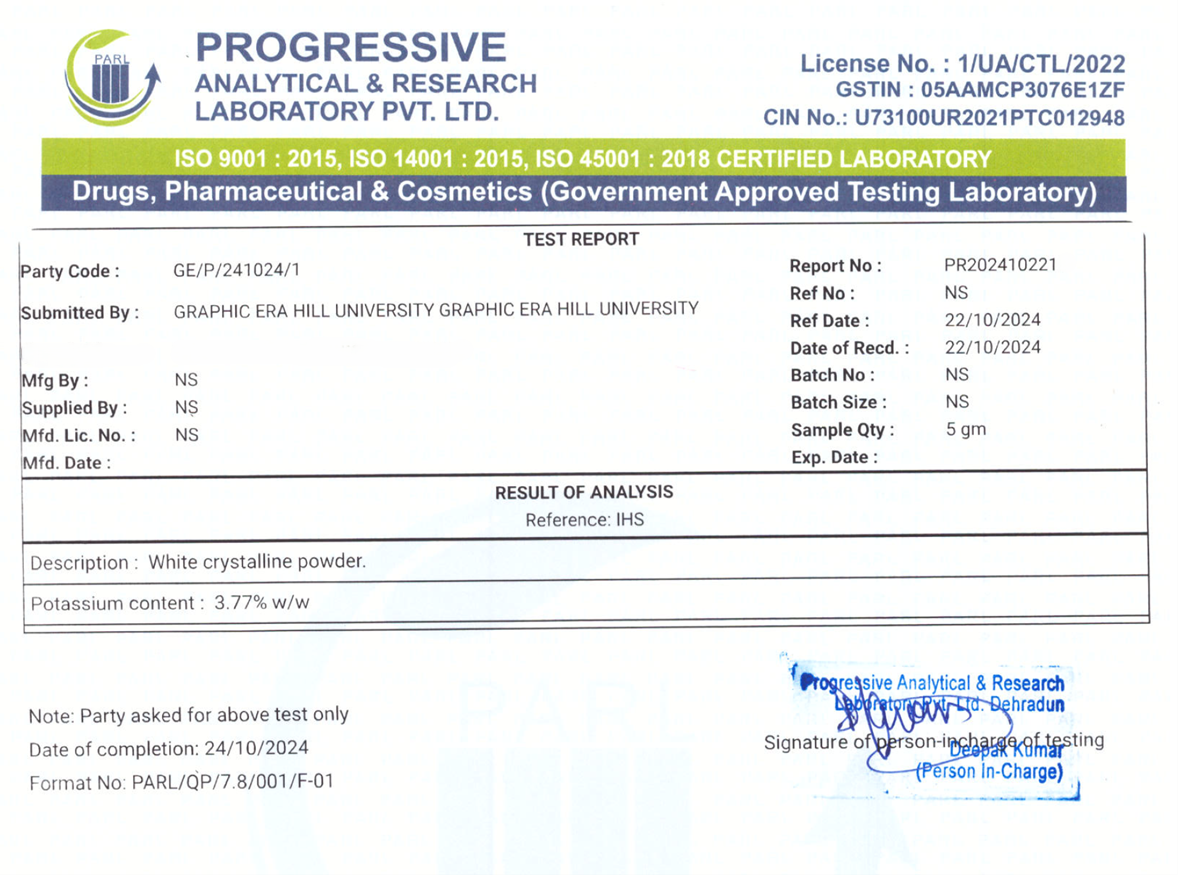


**Figure S1**: Atomic Absorption spectroscopy of the carrageenan gel

**Table S1: Result of the *In-vitro* Cell based safety:**

| **ref** | **Run1** | **Run2** | **Run3** | **mean** | **% Viability** | **% Inhibition** |
| --- | --- | --- | --- | --- | --- | --- |
| **0** | 0.447 | 0.443 | 0.445 | 0.445 | 100 | 0 |
| **6.25** | 0.438 | 0.437 | 0.433 | 0.436 | 97.97 | 2.03 |
| **12.5** | 0.432 | 0.432 | 0.429 | 0.431 | 96.78 | 3.22 |
| **25** | 0.418 | 0.419 | 0.417 | 0.418 | 93.93 | 6.07 |
| **50** | 0.409 | 0.407 | 0.408 | 0.408 | 91.75 | 8.25 |
| **100** | 0.397 | 0.395 | 0.393 | 0.395 | 88.73 | 11.27 |

**Table S2: Temp Vs Conductivity (**mS/cm)

| **Temperature (°C)** | **Conductivity 1** | **Conductivity 2** | **Conductivity 3** | **Mean** | **SD** |
| --- | --- | --- | --- | --- | --- |
| 21 | 8 | 8.8 | 9.6 | 8.8 | 0.8 |
| 26 | 8.4 | 9.4 | 10.4 | 9.4 | 1 |
| 31 | 9.3 | 10.3 | 11.3 | 10.3 | 1 |
| 36 | 10 | 11.2 | 12.4 | 11.2 | 1.2 |
| 41 | 11.6 | 12.3 | 13 | 12.3 | 0.7 |
| 46 | 12.6 | 13.2 | 13.8 | 13.2 | 0.6 |
| 51 | 15.8 | 16.3 | 16.8 | 16.3 | 0.5 |
| 56 | 17.6 | 18.2 | 18.8 | 18.2 | 0.6 |
| 61 | 19.1 | 20.1 | 19.1 | 19.4 | 0.6 |
| 66 | 19.9 | 21.1 | 22.3 | 21.1 | 1.2 |
| 71 | 21 | 22.2 | 23.4 | 22.2 | 1.2 |
| 76 | 20.9 | 22.4 | 23.9 | 22.4 | 1.5 |
| 81 | 21.4 | 22.4 | 23.4 | 22.4 | 1 |

| **Temperature** | **Conductivity (**mS/cm**)** |
| --- | --- |
| 21 | 8.8 ±0.8 |
| 26 | 9.4 ±1.0 |
| 31 | 10.3 ±1.0 |
| 36 | 11.2±1.2 |
| 41 | 12.3±0.7 |
| 46 | 13.2±0.6 |
| 51 | 16.3±0.5 |
| 56 | 18.2±0.6 |
| 61 | 20.1±1.0 |
| 66 | 21.1±1.2 |
| 71 | 22.2±1.2 |
| 76 | 22.4±1.5 |
| 81 | 22.4±1.0 |

- Weight desired amount of kappa carrageenan and dissolve it in desired volume of distilled water using a magnetic stirrer for 1 hour at 60^0^C.
- After 1 hour, allow it to cool to form gel.
- After the gel is formed, check its conductivity using a conductivity meter
- Now maintain the temperature of the gel at 37^0^C, and subsequently add more volume of water in ml.
- Again, dissolve it properly and maintain the temperature.
- Keep on adding water until we get a constant value.

**Kappa- carrageenan gel and NH4OH solution conductivity**

1. Weight 0.5gm kappa carrageenan and dissolve it in 65ml distilled water using a magnetic stirrer for 1 hour at 60^0^C.
2. After 1 hour, allow it to cool to form gel.
3. After the gel is formed, check its conductivity using a conductivity meter
4. Now maintain the temperature of the gel at 37^0^C, and subsequently add dropwise ammonium hydroxide (100µl).
5. After each drop, mix it in the gel and check the conductivity.
6. Lastly, plot a graph between volume of NH4OH (µl) in the kappa carrageenan gel v/s conductivity (mS).

**Control experiment**

- Add increasing volume of ammonium hydroxide to pure water and measure conductivity.
- This gives conductivity profile of ammonium hydroxide alone.
- Compare the conductivity measurements of ammonium hydroxide in water and with those in kappa CGN solution. This shows the interaction between kappa CGN and NH_4_OH.

**\**

**Table S3. Effect of NH₄OH concentration on KC gel conductivity.**

| **NH₄OH Conc. (µL)** | **Mean Conductivity (mS)** | **SD** | **Conductivity** | **Conductivity** | **Conductivity** |
| --- | --- | --- | --- | --- | --- |
| 100 | 9.1 | 1.2 | 7.9 | 9.1 | 10.3 |
| 200 | 9.1 | 1.1 | 8.0 | 9.1 | 10.2 |
| 300 | 8.8 | 0.8 | 8.0 | 8.8 | 9.6 |
| 400 | 8.8 | 1.6 | 7.2 | 8.8 | 10.4 |
| 500 | 8.5 | 1.0 | 7.5 | 8.5 | 9.5 |
| 600 | 8.5 | 0.9 | 7.6 | 8.5 | 9.4 |
| 700 | 8.3 | 1.2 | 7.1 | 8.3 | 9.5 |
| 800 | 8.3 | 1.3 | 7.0 | 8.3 | 9.6 |
| 900 | 8.3 | 0.7 | 7.6 | 8.3 | 9.0 |

| **Conc. of NH_4_OH (µl)** | **Conductivity (mS)** |
| --- | --- |
| 100 | 9.1±1.2 |
| 200 | 9.1±1.1 |
| 300 | 8.8±0.8 |
| 400 | 8.8±1.6 |
| 500 | 8.5±1.0 |
| 600 | 8.5±0.9 |
| 700 | 8.3±1.2 |
| 800 | 8.3±1.3 |
| 900 | 8.3±0.7 |

**
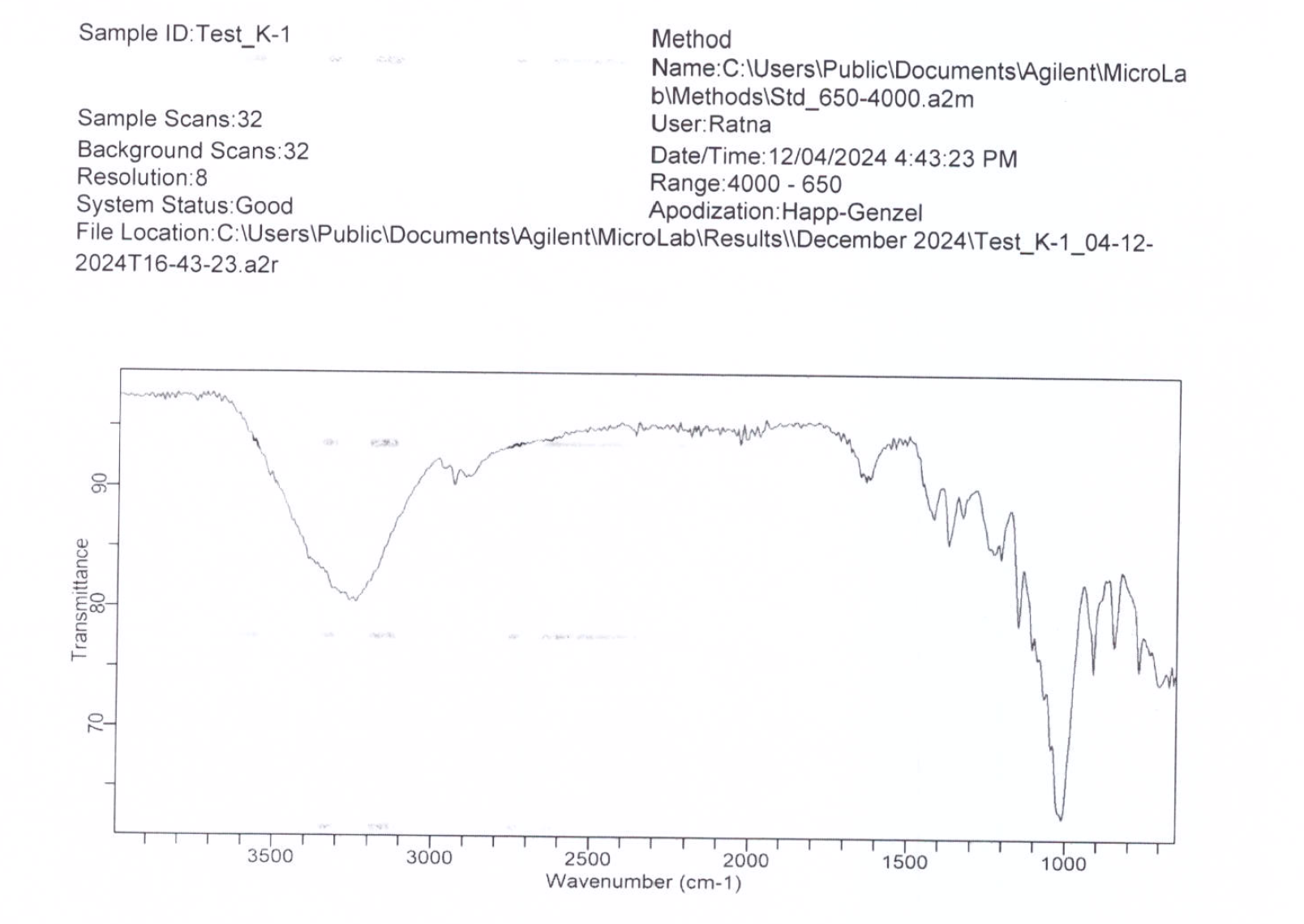
**

**
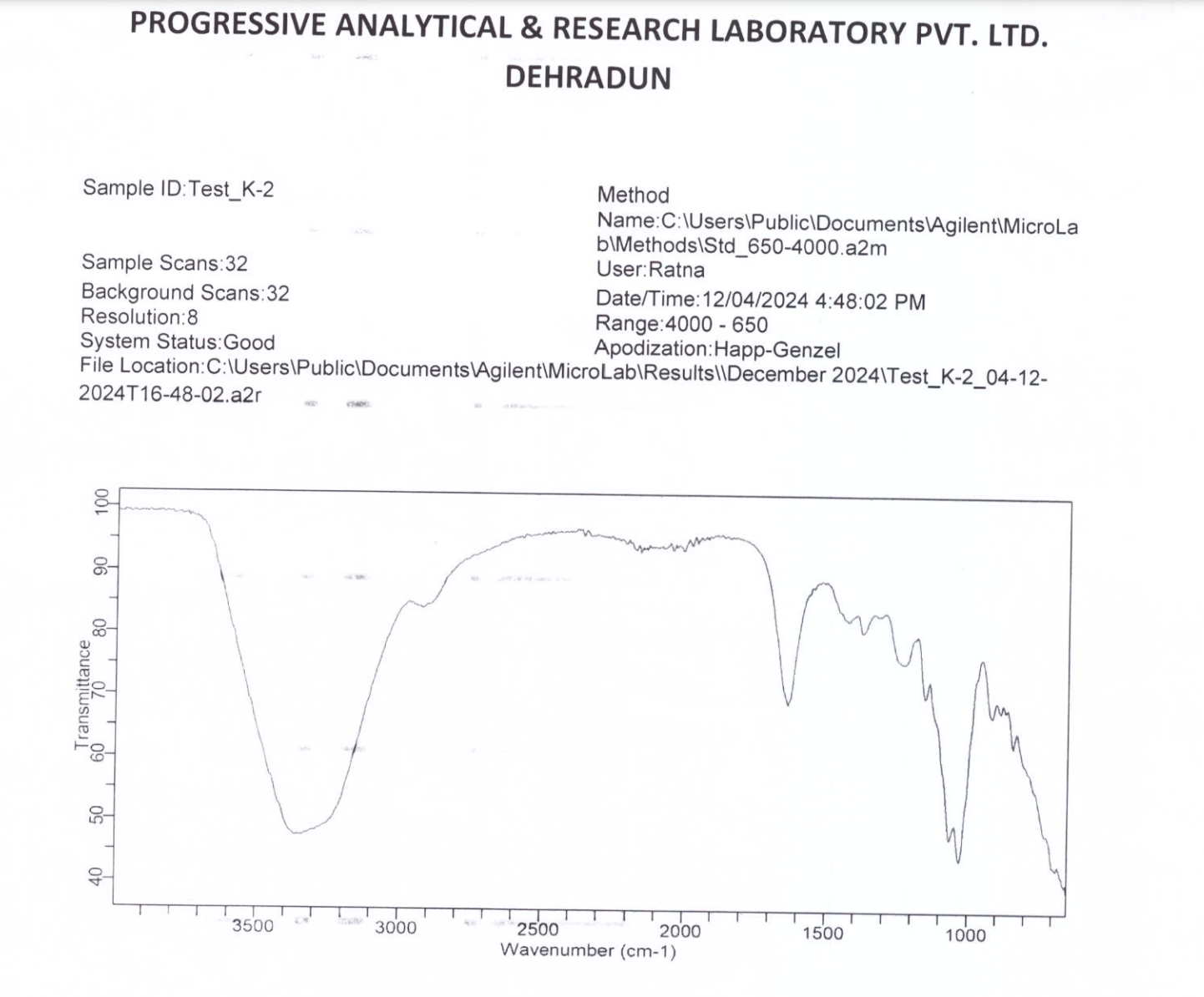
**

**Figure S3**: IR of the KC gel and NH4OH treated KC gel

**Table S4. FTIR Spectral Comparison of Blank KC Gel and NH₄OH-Treated KC Gel**

| Functional Group | Typical Assignment | Blank KC Gel (cm⁻¹) | NH₄OH-Treated KC Gel (cm⁻¹) | Observation |
| --- | --- | --- | --- | --- |
| –OH stretching | Intermolecular hydrogen bonding | ~3400 | Broadened at ~3425 | Hydrogen bonding increased after NH₄OH exposure |
| –SO₃⁻ asymmetric stretching | Sulfate ester groups | ~1250 | Reduced to ~1235 | Weakening due to sulfate–cation bond disruption |
| –C–O–C vibration | Galactose ring vibration / glycosidic linkage | ~1025 | Shifted to ~1015 | Slight change due to matrix loosening |
| –C–H bending | Aliphatic chain vibration | ~925 | ~920 | Minor shift, not structurally significant |
| –S=O symmetric stretching | Sulfonate groups | ~840 | Reduced to ~830 | Indicates rearrangement of ionic interactions |

***In Vitro* Drug Release Study & Kinetic Modelling**

This section provides raw experimental and model-fitted data supporting the drug release kinetics analysis of celecoxib from κ-carrageenan gels under blank and NH₄OH-treated conditions. It contains cumulative release data, kinetic parameters, and mathematical model details.

**Table S5. Calibration data for celecoxib quantification by UV spectrophotometry at 254 nm.**

| Concentration (mg/mL) | Absorbance (254 nm) |
| --- | --- |
| 0 | 0 |
| 2 | 0.12 |
| 4 | 0.24 |
| 6 | 0.36 |
| 8 | 0.49 |
| 10 | 0.62 |

**^Figure S4^** ^Standard calibration curve of celecoxib (0–10 mg/mL) at 254 nm^

**Raw experimental data for *in-vitro* drug release**

**Table S6. Comparative in vitro drug release profile of celecoxib from blank and NH₄OH-treated kappa-carrageenan (KC) gels.**

| Time (min) | Blank KC Release (%) | Treated KC Release (%) | Blank KC Release (mg) | Treated KC Release (mg) |
| --- | --- | --- | --- | --- |
| 0 | 0 | 0 | 0 | 0 |
| 15 | 4 | 12 | 0.4 | 1.2 |
| 30 | 10 | 28 | 1 | 2.8 |
| 45 | 17 | 45 | 1.7 | 4.5 |
| 60 | 24 | 63 | 2.4 | 6.3 |
| 90 | 28 | 74 | 2.8 | 7.4 |
| 120 | 30 | 81 | 3 | 8.1 |
| 180 | 33 | 86 | 3.3 | 8.6 |

Note: Values represent cumulative celecoxib release measured by UV spectrophotometry at 254 nm.

**Kinetic Modelling**

## **Table S7. Mathematical models applied for kinetic fitting.**

| **Model** | **Equation** | **Plot** | **Mechanism** |
| --- | --- | --- | --- |
| Zero Order | Q_t_ = k_0_t | Q_t_ vs. t | Constant release rate |
| First Order | log(100−Q_t_)=log100−(k_1_t/2.303) | log(100−Q_t_) vs. t | Concentration-dependent release |
| Higuchi | Q_t_ = k_H_ **_√_**t​ | Q_t_ vs. √t | Diffusion-controlled release |
| Korsmeyer-Peppas | Q_t_/Q_∞_= k_KP_ t ^n^ | log(Q_t_/_∞_)vs. logt | Diffusion and polymer relaxation (anomalous transport) |

**Table S8. Kinetic parameters for drug release models.**

| **Formulation** | **Model** | **Rate Constant (k)** | **Release Exponent**  **(n)** | **Correlation Coefficient**  **(R²)** |
| --- | --- | --- | --- | --- |
| **Blank KC Gel** | Zero-order | 0.189 %/min | – | 0.982 |
|  | First-order | 0.00233 min⁻¹ | – | 0.976 |
|  | Higuchi | 2.853 %·min⁻½ | – | **0.991** |
|  | Korsmeyer–Peppas | 0.487 | **0.85** | 0.985 |
| **NH₄OH-Treated KC Gel** | Zero-order | 0.463 %/min | – | 0.963 |
|  | First-order | 0.00401 min⁻¹ | – | 0.951 |
|  | Higuchi | 4.876 %·min⁻½ | – | 0.972 |
|  | Korsmeyer–Peppas | 0.612 | **0.68** | **0.974** |

Kinetic constants (k) and exponents (n) were derived from non-linear regression fits to zero-order, first-order, Higuchi, and Korsmeyer–Peppas models. The model with the highest correlation coefficient (r²) indicates the best fit.

**Detailed release data is provided in this attached excel file-**

**Binding Affinity and Molecular Docking**

**Methodology**

**Computational Docking Study**

Molecular docking studies were carried out to assess the binding affinity and interactions of κ-carrageenan (KC) and ι-carrageenan (IC) with key inflammatory targets, including Toll-like receptor 4 (TLR4), nuclear factor kappa B (NF-κB), and Janus kinase/signal transducer and activator of transcription (JAK/STAT) pathway proteins.

**Protein and Ligand Preparation**

The 3D crystal structures of TLR4 (PDB ID: 3FXI), NF-κB (PDB ID: 1NFI), and JAK/STAT (PDB ID: 6NJS) were obtained from the Protein Data Bank (PDB). To enhance docking accuracy, energy minimization and optimization of protein structures were performed using AutoDock Tools, which included removal of water molecules and the addition of polar hydrogen atoms. The 3D structures of KC and IC were developed using ChemDraw and further refined through energy minimization to ensure accurate docking simulations.

**Docking Protocol**

Molecular docking was performed using Auto Dock Vina to predict the binding interactions of κ-carrageenan and ι-carrageenan with the selected proteins. A grid box was defined around the active binding sites of each target protein based on literature-reported key residues. Lamarckian Genetic Algorithm was applied with default parameters to identify the most stable conformations based on binding energy (kcal/mol). The docking scores were analysed to compare the relative binding affinities of κ-carrageenan and ι-carrageenan for each target.

**Supplementary Table S9-** Molecular docking scores (kcal/mol) of KC and IC with inflammation-related targets (TLR4, NF-κB, IL-10). Lower scores indicate stronger binding affinities. (Data were generated by Swiss Dock online docking tool by using Auto Dock Vina method.)

| **S.No.** | **Target** | **Ligands** | **Binding affinity (Kcal/mol)** | **Main Binding sites** |
| --- | --- | --- | --- | --- |
| 1. | TLR4  (pdb id – 3FXI)  PDB doi:  <https://doi.org/10.2210/pdb3FXI/pdb> | Kappa  Carrageenan | -5.284 | GLN599, SER613, GLU605, PRO619, ASP580, ILE625, SER589, ASN409, PHE272 |
|  |  | Iota  Carrageenan | -7.354 | CYS585, PHE581, CYS542, ALA582, THR577, VAL338, ILE301, SER386, GLY249 |
| 2. | NF-κB  (pdb id- 1NFI)  PDB doi:  <https://doi.org/10.2210/pdb1NFI/pdb> | Kappa  Carrageenan | -7.005 | VAL268, THR316, GLU268, SER311, ARG304, SER234, LEU269, SER234, GLN267 |
|  |  | Iota  Carrageenan | -6.584 | TYR288, GLU288, GLU200, ALA158, GLU92, VAL299, ASP280, LYS314, GLU125 |
| 3. | JAK STAT  (pdb id- 6NJS)  PDB doi:  <https://doi.org/10.2210/pdb6NJS/pdb> | Kappa  Carrageenan | -5.127 | LYS631, THR663, PRO669, ILE628, ILE653, LEU608, ARG595, LEU608, TRP564, CYS550, SER319 |
|  |  | Iota  Carrageenan | -3.341 | LYS631, LEU577, ASP570, GLU594, PHE610, VAL338, GLN274, MET304, ALA475, ALA227, GLU434 |

This suggests that KC could contribute to immunosuppressive effects by stimulating IL-10 production, further supporting its superior anti-inflammatory activity observed in vivo.

**References:**

1. Akanksha Bhatt, Muskan Singh, Nikita Thapliyal, and **Purohit, Priyank***. "A feasible chemo preventive approach involves the use of zinc-coated curcumin with a carrageenan matrix for improved stability, solubility, and bioavailability" Journal of Polymer Engineering, 2025. <https://doi.org/10.1515/polyeng-2024-0066>
2. Akanksha Bhatt, **Priyank purohit,** Magda H. Abdellattif, Precision-engineered Carrageenan Gels: Boosting the Efficacy, Selectivity, and Release of Celecoxib for Lung Cancer Therapy, Anti-Cancer Agents in Medicinal Chemistry; Volume 25, Issue ,2025, e18715206376021.DOI: 10.2174/0118715206376021250506104129.
3. **Purohit P**, Mittal RK, Upadhyay PK, Bhatt A. Transforming Carbohydrates Through Multicomponent Reactions: Advances and Applications. Chemistry Select. **2025** Apr;10(15):e202406015.
4. Kumar, S., Bhatt, A. & Purohit, P. Carrageenan Modifications: Improving Biomedical Applications. J Polym Environ (**2025**).
5. Akanksha Bhatt, Surbhi Panwar, Priyank Purohit. Iota carrageenan, a Sustainable polymer transformed into a nanogel formulation, by using environment friendly method for antitumor action. Journal of Polymer and Composites. **2025**; 13(01):881-889. <https://doi.org/10.37591/JOPC.v13i01.188661>
6. Kailkhura, S., **Purohit, P**., Bhatt, A. et al. Eclipsed conformational locking: exploring iota carrageenan’s distinct behavior in ethanol–water systems via hydrogen bonding with the disulfate group. Chem. Pap. (2024). https://doi.org/10.1007/s11696-024-03590-4.
7. A. Bhatt, S. Kailkhura, A. Shukla, S. Kumar, P. Purohit, M H. Abdellattif, Modulating Ionic Linkages in the Heterocyclic Sulfated Polysaccharide Carrageenan for Enhanced Selectivity Against Amelanotic Melanoma Cells. *Chemistry Select*, May 2024, 9, 20, <https://doi.org/10.1002/slct.202400185>
8. Shukla, A., Kumar, S., Bhatt, Purohit Priyank*.* Conversion of iota carrageenan hydrocolloids to hydrophobic hydrocolloids, by the replacement of potassium to barium ion, for the entrapment of water insoluble drugs. *Discov Appl Sci* **6**, 244 (2024). <https://doi.org/10.1007/s42452-024-05925-y>
9. A. Bhatt, S. Kailkhura, P. Purohit Benzoylation of Iota Carrageenan: Development of a Stable, Conductive, and Hydrophobic Drug Carrier with Reduced Toxicity and Improved Gel-Forming Ability. *Macromol. Chem. Phys*. 2024, 2400017. https://doi.org/10.1002/macp.202400017
